# Magnetism in Au-Supported Planar Silicene

**DOI:** 10.3390/nano11102568

**Published:** 2021-09-29

**Authors:** Mariusz Krawiec, Agnieszka Stępniak-Dybala, Andrzej Bobyk, Ryszard Zdyb

**Affiliations:** 1 Institute of Physics, M. Curie-Sklodowska University, Pl. M. Curie-Skłodowskiej 1, 20-031 Lublin, Poland; agnieszka.stepniak@umcs.pl (A.S.-D.); zdybr@hektor.umcs.lublin.pl (R.Z.); 2 Institute of Computer Science, M. Curie-Sklodowska University, ul. Akademicka 9, 20-031 Lublin, Poland; andrzej.bobyk@mail.umcs.pl

**Keywords:** magnetism, 2D materials, silicene, Fe, Co, Ab initio

## Abstract

The adsorption and substitution of transition metal atoms (Fe and Co) on Au-supported planar silicene have been studied by means of first-principles density functional theory calculations. The structural, energetic and magnetic properties have been analyzed. Both dopants favor the same atomic configurations with rather strong binding energies and noticeable charge transfer. The adsorption of Fe and Co atoms do not alter the magnetic properties of Au-supported planar silicene, unless a full layer of adsorbate is completed. In the case of substituted system only Fe is able to produce magnetic ground state. The Fe-doped Au-supported planar silicene is a ferromagnetic structure with local antiferromagnetic ordering. The present study is the very first and promising attempt towards ferromagnetic epitaxial planar silicene and points to the importance of the substrate in structural and magnetic properties of silicene.

## 1. Introduction

The great success of graphene [1,2] has triggered research into exploring alternative two-dimensional (2D) Dirac materials [3,4,5,6]. Among them, the group-14 2D graphene followers: silicene (Si), germanene (Ge), stanene (Sn), and plumbene (Pb), also known as 2D-Xenes [6,7,8,9], have attracted extensive interests. The crystal lattice of each freestanding Xene is predicted to be buckled on the atomic scale, and can be viewed as a structure with two sublattices displaced vertically as a result of the pseudo-Jahn-Teller (PJT) distortion [10]. Nevertheless, the electronic band structure in the form of Dirac cones is preserved in Xenes too.

A considerable effort has been made to synthesize silicene. Some attempts have succeeded and silicene has been grown in the form of a lattice on a substrate on a few templates [11,12,13,14,15,16,17,18,19,20,21,22]. Usually the epitaxial silicene displays a variety of different phases or surface reconstructions. However, when the silicene-substrate interaction is not much destructive, another allotropic phase of silicene, the planar form, can be obtained. The free-standing planar silicene is dynamically unstable [23]. However, presence of the substrate can effectively suppress the PJT effect, and the planar supported form of silicene can exist [10,24,25]. This has been demonstrated in the case of silicene prepared on Au(111) films grown on Si(111) [21,26] and on mica substrates [27].

From the very beginning silicene has been intensively researched as a promising candidate for the next-generation nanoelectronics beyond current metal-oxide-semiconductor technology [6,28]. Indeed, the silicene-based field effect transistor has been proposed [29,30] and demonstrated to work at room temperature [31,32]. Moreover, 2D materials in general, and silicene in particular, serve as a promising platform to exploit also various spin-related phenomena. The exploration of the spin degree of freedom of an electron brings more functionalities to these materials in view of novel, low-power, high-performance spintronic devices [33,34,35,36,37,38,39,40,41]. However, the realization of room-temperature 2D ferromagnetism is a big challenge. In particular, graphene and other Xenes fail to exhibit strong magnetism due to the delocalization of s and p orbitals. To think about spintronic applications they must be functionalized. Indeed, a remarkable progress in 2D magnetism in Xenes has been made, mainly via chemical dopants, defects, engineering of edges, and proximity of magnetic layers [38,39,41]. Perhaps the most natural way of developing robust magnetism in Xenes is to decorate their 2D sheets by transition metal (TM) atoms known for strong ferromagnetic behavior.

In this work, motivated by recent experimental realization of epitaxial planar silicene [21,26], we explore structural, energetic and magnetic properties of Fe- and Co-doped planar silicene/Au structure by using first-principles density functional theory (DFT) calculations. We show that very promising in view of the formation of 2D ferromagnets is Fe-substituted silicene, which features the strong ferromagnetism with a local antiferromagnetic ordering.

## 2. Methods

First-principles density functional theory calculations were performed within spin-polarized Perdew-Burke-Ernzerhof [42] generalized gradient approximation to the exchange-correlation interaction, as implemented in VASP (Vienna ab-initio simulation package) [43,44]. The core electrons were treated within the projector-augumented wave method [45]. The plane-wave energy cutoff for all calculations was set to 350 eV. The convergence criterion of total energy for self-consistent field calculations was chosen to be 10${}^{-7}$ eV.

The silicene/Au(111) structure has been modeled by a freestanding AuSi${}_{2}$ system in which Au atoms have been placed below a freestanding silicene layer. The atomic positions were relaxed by a conjugate gradient method until the largest force in any direction was below 0.001 eV/Å. To mimic epitaxial form of silicene, the lattice constant and vertical positions of Au atoms were kept fixed during calculations for all the considered structures. A vacuum layer of 17 Å was added to avoid periodic interactions.

Supercell models consisting of an m×m unit silicene cell (m= 1, 2, 3, 4, and 5) with one metal (M = Fe, Co) atom were studied. The M-adsorbed Au-supported planar silicene is denoted as MAu${}_{n}$Si${}_{2n}$, where $n=m^{2}$, while substituted structure—MAu${}_{n}$Si${}_{2n-1}$. The surface doping concentrations of adsorbed and substituted systems are defined as $N={\left(2n\right)}^{-1}\times 100$%. The Brillouin zone of 1×1 structure was sampled by a 16×16×1 Monkhorst-Pack k-points grid including the Γ point [46]. The k-points grid was adjusted according to the size of the unit cell.

Spin-orbit (SO) interaction was not included in the calculations, although it may be significant for the Co ions, in particular, in the context of topological properties [47,48]. It was predicted that the SO coupling together with not too strong induced magnetic moments can make favorable conditions for the realization of quantum anomalous Hall effect in Co/silicene [48].

## 3. Results and Discussion

**Figure 1:**
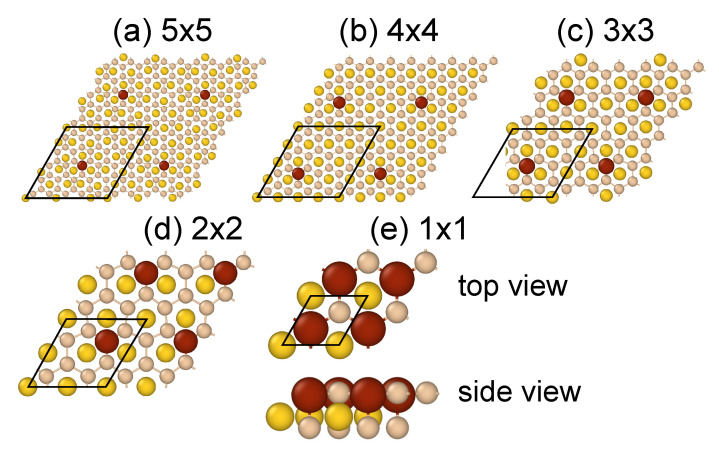
Structures of Fe-covered Au-supported planar silicene with a coverage of N=2.0% (**a**), 3.1% (**b**), 5.6% (**c**), 12.5% (**d**), and 50.0% (**e**). Unit cells for each structure are marked by black rhombi.

The optimized atomic structure of supported planar silicene modeled by the AuSi${}_{2}$ system has the lattice constant of 4.10 Å and Si-Au vertical separation of 1.30 Å, which are in good agreement with previous study [21]. Due to the planar form of supported silicene only a single substitutional and three possible adsorption sites, top (T), bridge (B), and hollow (H), were investigated. The H site is right above the center of a silicene hexagonal ring, and coincides with the top site with respect to Au. The T site is above a Si atom, and the B site is above the middle of the Si-Si bond. The top site is the most favorable site for both metals at all considered concentrations of their atoms, Figure 1.

This is the first substantial difference with respect to previous studies concerning the adsorption of Co and Fe on a freestanding silicene, where the most preferable site was the hollow site [47,49]. Although a slightly more stable adsorption site for Fe found in Ref. [50] was the top site above a Si atom of sublattice A (TA). In the present case adsorbed atoms try to build into silicene lattice by expelling underneath Si atoms. This is visible in Figure 1e and reflected in the bonding characteristics. The adsorbed atoms form the shortest bonds with the underneath Si atoms, $d_{MSi,v}$ = 2.17–2.22 Å for Co, and 2.12–2.29 Å for Fe, depending on the coverage *N*. At the same time bond lengths between adatoms and the in-plane Si atoms, $d_{MSi,h}$ yield 2.23–2.37 Å for Co, and 2.26–2.37 Å for Fe, respectively. Lengths of the M-Si bonds, in particular $d_{MSi,h}$ agree well with previous study concerning the adsorption on a free-standing silicene [50], similar as the fact of the overall slightly shorter Fe-Si bonds than Co-Si bonds. There is no such difference in the case of M-Au bonds. Both adsorbates form bonds of the same length. The structural parameters of Au-supported planar silicene with adsorbed M atoms are summarized in Table 1.

Despite the formation of bonds between adsorbates and the substrate, also substrate itself undergoes the local deformation. The strongest deformation, characterized by the thickness of the silicene/Au structure, Δz, takes place at the lowest adatom coverage, as Table 1 shows. Obviously this is related to the size of the unit cell and periodic boundary conditions.

It is natural to expect some correlation between length of the bonds and the adsorption energy. In general, the longer the bond the higher the binding (or adsorption) energy. The present systems confirm this common chemical wisdom, see Table 1. Furthermore, it is worth noting that at surface coverages less than 50% the bond-energy correlation is governed by M-Si bonds, as the adatoms form with Au atoms equally-long bonds. This is strictly related to the local deformation of atomic structure. At extremely high coverage, the deformation of silicene/Au structure suppresses, and both Au- and Si-M bonds elongate. Similar, as in the case of the adsorption on a free-standing silicene, Co atoms bind more strongly than Fe [49,50]. However, in the present system, due to bonds formed with Au atoms, the adsorbed energy becomes higher than in the free-standing case. For example, at 3.1% surface coverage, the energy increases by 1.7 eV for Co, and by 1.2 eV for Fe adatoms [50].

Bonding of Fe and Co adatoms to silicene/Au is accompanied by a noticeable charge transfer. Both adsorbates receive electrons from the substrate, as the Bader charge analysis shows, Table 1. The electron gain yields 0.5–0.6 e for Co, and 0.3–0.4 e for Fe, and points to the partially ionic character of bonding. The electron charge comes mainly from the underneath expelled Si atoms and to a lesser extent from the neighboring Si atoms. Au atoms also are negatively charged, as each Au atom receives ~0.46 e from Si layer. Exactly the same value was found in the case of Au-supported planar silicene [21], which points to no charge transfer between Fe/Co and Au atoms, and suggests the formation of covalent bonds between these species. It is worth noting that the adsorption of Fe and Co on a free-standing silicene does not induce charge transfer between adsorbate and silicene [49].

Considerable negative doping of adsorbate atoms prohibits the formation of magnetic order in the system. It is known that magnetism in transition metal materials is related to partially filled *d* shells. The isolated transition metal atoms behave like small magnets unless their *d* shells are completely filled. Thus it is natural to expect the suppression of the magnetism in the present case. Nevertheless, the ferromagnetic state can appear at extremely high surface coverage, N=50%, which in fact corresponds to a full single Fe or Co layer. In this case the charge transfer is strongly suppressed, almost ten times for Fe, and twice for Co. The calculated corresponding magnetic moments yield 2.18 and 0.24 $\mu_{B}$, respectively, and are comparable to the case of the free-standing silicene, albeit at lower surface coverage [50].

Considering a simple exchange model which relates the splitting of quasilocal 3d states of M atoms and the net magnetic moments, one can estimate the effective exchange coupling $J_{eff}$ [51,52]. The calculated splitting of main peaks of the band structure projected on 3d states of Fe and Co atoms are found to be 1.17 eV and 0.12 eV, respectively. This results in corresponding values of the $J_{eff}$ equal to 2.1 eV (Fe) and 2.0 eV (Co), which gives the mean-field values of the Curie temperature of the order of 6000 K.

**Figure 2:**
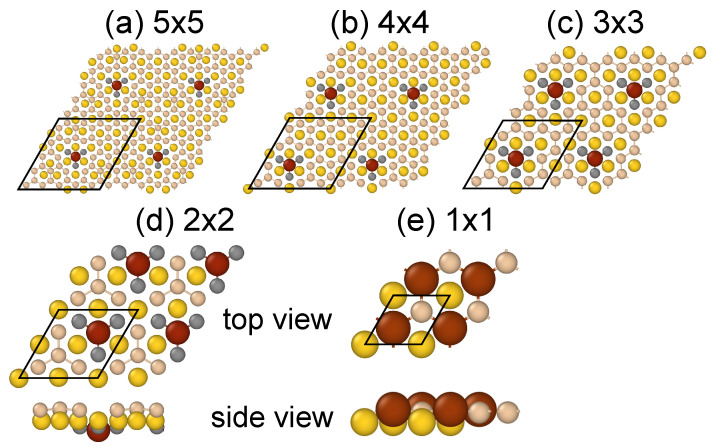
Atomic structures of Fe-substituted planar silicene/Au with a concentration of N=2.0% (**a**), 3.1% (**b**), 5.6% (**c**), 12.5% (**d**), and 50.0% (**e**). Unit cells for each structure are marked by black rhombi.

Since adsorbed Fe and Co adatoms tend to build into silicene lattice, it is natural to study the substitutional doping. Figure 2 displays atomic structures of Fe-substituted Au-supported planar silicene at different concentration of Fe atoms. Corresponding Co-related structures are almost identical.

A detailed inspection of the structural models reveals that M atoms go below the Au layer in the proces of geometry optimization, except the case of the highest concentration N=50%, see Figure 2d,e and values of $h_{MAu}$ in Table 2. The M atoms reside ~1 Å below the Au layer and are surrounded by three Si atoms, as it is demonstrated in Figure 2d. The situation is identical in larger unit cells; only M atom and three neighboring Si atoms go below the Au layer, and the remaining atoms stay on-top of it. Other structural parameters listed in Table 2, like lengths of M-Au $d_{MAu}$ and M-Si bonds $d_{MSi}$ or thickness of the structure Δz are very close to the corresponding values in Table 1. This is in line with conclusions drawn from studying the adsorbate systems that Fe and Co atoms prefer to be built into the silicene lattice.

Similar as previously, Fe atoms are slightly weakly bound to the silicene/Au than Co atoms. The binding energies of substituted structures vary between 5 and 8 eV, and are of the same order as in the case of the adsorbed systems. They are even almost identical for the highest concentration structures. However, as the concentration of M atoms decreases the binding energies of the substituted systems become higher. This suggests that the substitution is preferable over the adsorption. The energetics confirms this scenario except the extreme, N=50%, coverage structures. The total energy difference between the adsorption and the substitution phase of Fe varies between −0.6 and +0.5 eV. The same trend is observed for Co with energies changed between −0.7 and +0.4 eV. However, this does not mean that substitution phases will be preferred at normal conditions, as the energy barriers for substitution process remain unknown. Therefore the annealing of the sample might be required.

Somehow counterintuitive picture is observed in the charge transfer process. One could expect that Fe and Co atoms, having more valence electrons than Si, should act as n-doped impurities in the Si lattice. While this is true for Fe atoms, Co atoms exhibit just opposite behavior, as Table 2 shows. The charge transfer, in particular the direction of electron flow, is strictly related to the magnitude of the total magnetic moments, thus to the formation of the ferromagnetic order in the system. Co atoms receive electrons mainly from neighboring Si atoms, fill their *d*-shells and suppress the magnetism, similar as in previously discussed adsorbed Co atoms on silicene/Au. On the other hand, Fe atoms strengthen their magnetic properties by emptying their *d*-shells, which is visible even at very low concentration of Fe atoms. Note, that the ferromagnetic order is present in all studied here Fe-substituted system. In this regard, the Co-doped silicene/Au is very similar to the Co-adsorbed structure.

The calculated values of the effective exchange coupling in the Fe-substituted structures yield: 3.2, 3.4, 3.0, 3.4, and 3.0 eV for concentrations N=2.0, 3.1, 5.6, 12.5, and 50%, respectively. A little lower value, 2.3 eV, has been found for Co-substituted system at N=50%. Note that these exchange coupling values are only slightly higher than in the case of the adsorbed systems.

**Figure 3:**
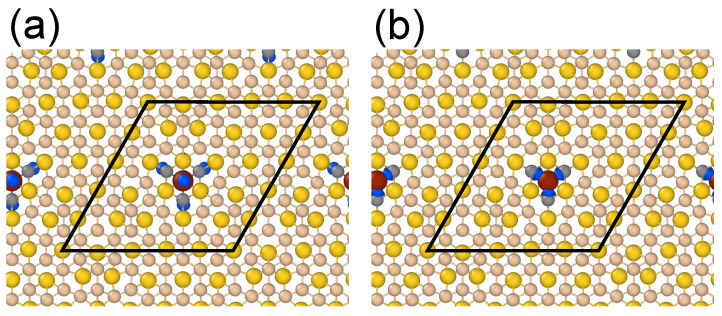
Spin density distribution of Fe-doped silicene/Au structure at concentration of Fe atoms N=2%. Blue isosurfaces represent positive (**a**) and negative (**b**) spin densities equal to $10^{-6}$ e/Å${}^{3}$, respectively.

Although major magnetism in Fe-doped planar silicene/Au structure is contributed by Fe atoms, neighboring Si atoms also participate in the formation of magnetic ground state. Figure 3 shows the calculated local spin density distribution.

The presence of Fe atoms trigger local antiferromagnetic (AFM) ordering. Starting from Fe atoms with positive spin density, the neighboring Si atoms are negatively spin polarized, while the next neibghor Si atoms share the same sign of the spin polarization with Fe. However, the magnetization pattern seems to be more complicated and unrelated to silicene sublattices. Figure 3 clearly shows that Si bonds become spin-polarized and they are mainly responsible for the local AFM order. This agrees well with findings of Ref. [52], where the broken π bonds of silicene were found to be responsible for the AFM ordering in hydrogen-decorated freestanding silicene.

Although the present AuSi${}_{2}$ model reproduces main features of the planar epitaxial silicene of Ref. [21], it has certain drawbacks when the adsorption or substitution of foreign atoms is considered. As it was already discussed the adsorbed M atoms push down the underneath Si atoms. In real situation the pushed Si atoms will feel the resistance of the substrate. Here, they go below the Au layer, which is an underisable effect in the present context. To check the impact of pushed down Si atoms on properties of the system, we performed additional calculations with constrained geometry, in which z-coordinates of these atoms were forced to be equal to z-coordinates of Au atoms. Of course, such constrainted atomic structures feature higher total energy, usually less than 1 eV for both adsorbate metals. However, we arrived at the same conclusions regarding magnetic properties of the system. Both adsorbate structures remain nonmagnetic, unless the concentration of adatoms is extremely high. In this case, i.e. for N=50%, magnetic moments decrease by 0.48 and 0.24 $\mu_{B}$ for Fe and Co, respectively.

A similar problem appears in the substitution phase, where M atoms together with three Si atoms go below the Au layer in the process of the geometry optimization. In this case we have applied a slightly different strategy. We fixed all coordinates of the Au atoms to block movement of M atoms through the Au layer. Same as before, the conclusions remain true. In particular, the Co-doped structures are nonmagnetic, while Fe-doped—magnetic, albeit with modified values of magnetic moments. They depend on the concentration of adatoms *N*, and grow with *N* from almost 0 for N=2% to 0.65 $\mu_{B}$ for N=12.5%. Note that when N=50%, the M atoms remain above the Au layer, see Figure 2e.

Finally we would like to comment on the validity of the approximation used in calculations. It is well known that Fe and Co are examples of strongly correlated materials, and the DFT calculations on GGA-PBE level may not fully correctly capture the physics of related systems. However, it was shown that the effect of the Hubbard U (GGA+U approach) does not change much the geometry of the Fe- and Co-decorated silicene as compared to the GGA-PBE [47]. Still the hollow sites remain the lowest energy adsorption sites, and only adsorbate-silicene bonds slightly elongate. Fortunately, the U almost does not affect the electron distribution, therefore the magnetic moments either.

Experimentally, it is still a big challenge to obtain 2D ferromagnetic layers. The present system does not seem to be unique in this respect, although somehow promising. To make the planar silicene/Au structure magnetic, the simple deposition of Fe or Co atoms and their adsorption may not be enough. The silicene/Au with adsorbed Fe or Co atoms is nonmagnetic unless a full layer of adsorbate is formed. It is necessary to substitute some Si atoms by Fe atoms, which should be achievable by the deposition on a hot substrate or annealing the already adsorbed system.

## 4. Conclusions

In conclusion, we presented a first-principles investigation of the adsorption and substitution characteristics of transition metals (Fe and Co) on Au-supported planar silicene. We focused on structural, energetic and magnetic properties of the system. Both dopants favor the same adsorbed and substituted configurations with rather strong binding energies and noticeable charge transfer. The adsorption of Fe and Co atoms do not alter the magnetic properties of Au-supported planar silicene, unless a full layer of adsorbate is completed. In the case of substituted system only Fe is able to produce magnetic ground state. The Fe-doped Au-supported planar silicene is a ferromagnetic structure with local antiferromagnetic ordering. The present study is the very first and promising attempt towards ferromagnetic epitaxial planar silicene and points to the importance of the substrate in structural and magnetic properties of silicene.

## Figures and Tables

**Table 1:** Calculated structural and energetic parameters for Au-supported planar silicene with adsorbed Fe and Co at their different coverages *N*: the height of the adatom over the Au plane ($h_{MAu}$), the bond length between the adatom and its nearest Au atoms ($d_{MAu}$), the bond length between the adatom and its nearest Si atoms in the plane ($d_{MSi,h}$), the bond length between the adatom and the underneath Si atom ($d_{MSi,v}$), thickness of the silicene/Au structure defined as the difference between the largest and smallest *z* coordinates of Si and Au atoms (Δz), the binding energy of adatoms on silicene/Au ($E_{b}$), charge transferred from silicene to adatom ($\Delta \rho_{M}$), total magnetic moment of the system ($\mu_{tot}$) in units of Bohr magneton ($\mu_{B}$).

Atom	*N* (%)	$h_{\mathrm{MAu}}$ (Å)	$d_{\mathrm{MAu}}$ (Å)	$d_{\mathrm{MSi},h}$ (Å)	$d_{\mathrm{MSi},v}$ (Å)	Δz (Å)	$E_{b}$ (eV)	$\Delta \rho_{M}$ (e)	$\mu_{\mathrm{tot}}$ ($\mu_{B}$)
Fe	2.0	0.87	2.64	2.32	2.12	3.15	6.246	0.33	0.00
	3.1	1.10	2.64	2.26	2.18	2.74	5.722	0.35	0.00
	5.6	1.03	2.64	2.27	2.16	2.75	5.509	0.37	0.00
	12.5	1.09	2.64	2.27	2.18	2.76	5.530	0.34	0.00
	50.0	1.52	2.81	2.37	2.29	2.44	4.925	0.02	2.18
Co	2.0	1.09	2.62	2.27	2.17	2.89	6.922	0.48	0.00
	3.1	1.23	2.64	2.23	2.22	2.84	6.443	0.58	0.00
	5.6	1.14	2.64	2.25	2.18	2.76	6.256	0.58	0.00
	12.5	1.16	2.64	2.27	2.20	2.77	6.164	0.52	0.00
	50.0	1.50	2.81	2.37	2.22	2.35	5.507	0.32	0.24

**Table 2:** Structural and energetic parameters for Fe- and Co-substituted silicene/Au system at their different concentrations *N*: the *z* coordinate of the M atom with respect to the Au plane ($h_{MAu}$), the bond length between the M atom and its nearst Au atoms ($d_{MAu}$), the bond length between the M atom and its nearest Si atoms ($d_{MSi}$), thickness of the silicene/Au structure defined as the difference between the largest and smallest *z* coordinates of Si and Au atoms (Δz), the binding energy of the M atom ($E_{b}$), charge transferred from silicene to the M atom ($\Delta \rho_{M}$), total magnetic moment of the system ($\mu_{tot}$) in units of Bohr magneton ($\mu_{B}$).

Atom	*N* (%)	$h_{\mathrm{MAu}}$ (Å)	$d_{\mathrm{MAu}}$ (Å)	$d_{\mathrm{MSi}}$ (Å)	Δz (Å)	$E_{b}$ (eV)	$\Delta \rho_{M}$ (e)	$\mu_{\mathrm{tot}}$ ($\mu_{B}$)
Fe	2.0	−1.22	2.70	2.29	2.77	7.410	−0.23	2.82
	3.1	−1.35	2.67	2.34	2.60	6.874	−0.33	2.45
	5.6	−1.26	2.69	2.29	2.65	5.847	−0.23	2.18
	12.5	−1.02	2.68	2.31	1.92	6.341	−0.26	1.55
	50.0	0.91	2.54	2.38	0.69	4.951	−0.34	2.40
Co	2.0	−1.05	2.66	2.21	2.82	7.929	0.18	0.00
	3.1	−1.18	2.63	2.26	2.58	7.318	0.07	0.00
	5.6	−1.21	2.68	2.22	2.67	6.438	0.17	0.00
	12.5	−1.00	2.68	2.25	1.91	6.896	0.11	0.00
	50.0	1.03	2.59	2.40	0.67	5.489	−0.09	0.52

## Data Availability

The data is available on reasonable request from the corresponding author.

## Abbreviations

The following abbreviations are used in this manuscript:

2D	Two-dimensional
PJT	Pseudo-Jahn-Teller
DFT	Density Functional Theory
SO	spin-orbit
AFM	antiferromagnetic
